# Molecular mechanism of ChaiShi JieDu granule in treating dengue based on network pharmacology and molecular docking: A review

**DOI:** 10.1097/MD.0000000000036773

**Published:** 2023-12-29

**Authors:** Cong Li, Luping Lin, Yexiao Tang, Sanqi Huang

**Affiliations:** a Guangzhou Eighth People’s Hospital, Guangzhou Medical University, Guangzhou, China.

**Keywords:** ChaiShi JieDu granule, CSJD, dengue, molecular docking, network pharmacology

## Abstract

Dengue fever is a frequently occurring infectious disease caused by the Dengue virus, prevalent in tropical and subtropical regions. Chaishi Jiedu Granules (CSJD) is an empirical prescription of the Eighth Affiliated Hospital of Guangzhou Medical University in the treatment of dengue fever, which has been widely used in the treatment of dengue fever, and has shown good efficacy in improving the clinical symptoms of patients. This study aims to explore the molecular mechanism of CSJD in treating dengue fever using network pharmacology, molecular docking techniques, and virtual screening methods. The results showed that luteolin, quercetin and other compounds in CSJD could target important targets related to dengue virus, including STAT3, AKT1, TNF, IL-6, and other key genes, thus playing an antiviral role. Among them, luteolin and wogonin in CSJD also inhibited dengue virus replication and reduced inflammation, and showed good binding force with IL-6 and TNF. Therefore, this study provides an important reference for the development of CSJD as a potential drug for dengue fever treatment and a new perspective for research and development in this field.

## 1. Introduction

Dengue fever is an acute infectious disease caused by the dengue virus (DENV), which spreads through bites from infected Aedes aegypti or Aedes albopictus mosquitoes.^[1]^ Dengue fever is mainly prevalent in tropical and subtropical countries worldwide, and its incidence has been increasing with global warming.^[2]^ There have been outbreaks of Dengue fever in some areas of China. Dengue fever can be classified as either mild or severe, with as many as 1 in every 20 patients developing severe dengue, which can lead to the risk of bleeding, shock, and even death.^[3]^ The global public health system is faced with a significant challenge due to the burden of dengue fever. There is currently no specific antiviral drug for Dengue fever, and clinical treatment mainly involves symptomatic and supportive care as well as prophylactic treatment. The first approved vaccine, Dengvaxia, faces the drawbacks of low efficacy and high side effects.^[4]^ However, studies have shown that traditional Chinese medicine has certain characteristics and advantages in treating Dengue fever, especially in reducing symptoms such as fever, headache, and muscle and joint pain, and also reducing the occurrence of severe Dengue fever.^[5]^

ChaiShi JieDu granule (CSJD) is an empirical prescription developed by the Eighth Affiliated Hospital of Guangzhou Medical University. Based on the traditional Chinese medicine concept of “heat warming and dampness suppressing, and qi and blood being the same disease,” CSJD is a Chinese herbal preparation primarily used to treat the fever stage of non-severe dengue fever. CSJD contains a blend of twelve high-quality ingredients, including gypsum, Anemarrhenae Rhizoma, Scutellariae Radix, Radix Bupleuri, Arum Ternatum Thunb, Radix Puerariae, talc, buffalo horn, Codonopsis Radix, Siphonostegiae Herba, Coicis Semen and licorice. Since 2014, CSJD has been clinically applied in China and Southeast Asian countries such as Cambodia and Pakistan.^[5]^ Clinical studies have shown that this prescription can effectively reduce fever and relieve pain. When combined with conventional treatment, it can shorten the fever duration of dengue patients, alleviate systemic symptoms such as headache, joint pain, and fatigue, and improve the overall therapeutic efficacy. Moreover, CSJD can reduce the rate of severe illness and shorten the length of hospital stay.^[6]^

Network pharmacology is a new research method emerging in recent years, which was first proposed by Professor Hopkins of pharmacology in the University of Dundee.^[7,8]^ Based on the theory of systems biology, this method is a new subject for multi-target-drug molecule design by selecting specific signal nodes. In order to improve the therapeutic effect of drugs, reduce toxic side effects, improve the success rate of new drug clinical trials, and save the cost of drug research and development, network pharmacology emphasizes the multi-channel regulation of targets on signaling pathways. Hopkins developed the concept of network pharmacology in 2007, based on the high rate of clinical failure of new drugs in the past due to “1 drug, 1 target, 1 disease.”^[9]^ The basic idea is to analyze the intervention and influence of drugs on the disease network based on the disease-gene-target-drug interaction network, so as to help people understand the pathological basis of disease and the therapeutic effect of drugs more comprehensively. Network pharmacology is mainly used to screen active ingredients, predict the target of action in ingredients, and study the mechanism of action of drugs. In addition, the prediction of multi-target and multi-pathway of compatible prescriptions by network pharmacology can confirm the concept of “treating different diseases with the same disease” in Traditional Chinese Medicine (TCM) theory. As a new research idea, network pharmacology can also be used to explain the compatibility of TCM compounds or to find new indications of TCM.^[10]^ Since traditional Chinese medicine is characterized by multiple ingredients, multiple targets, multiple pathways and overall regulation, and network pharmacology can be applied to the study of the pharmacodynamic substances and mechanism of traditional Chinese medicine, to explain the compatibility rules of traditional Chinese medicine compounds or to find new indications of traditional Chinese medicine, the combination of traditional Chinese medicine and network pharmacology will become a new method for the study of traditional Chinese medicine.^[9]^ The characteristics of complex networks are reflected in TCM from clinical syndromes to prescriptions. Soon after the concept of network pharmacology was put forward, it was accepted and widely applied by the Chinese medicine circle. Its overall advantages provide a new idea for the study of complex TCM system.

**Figure 1.**
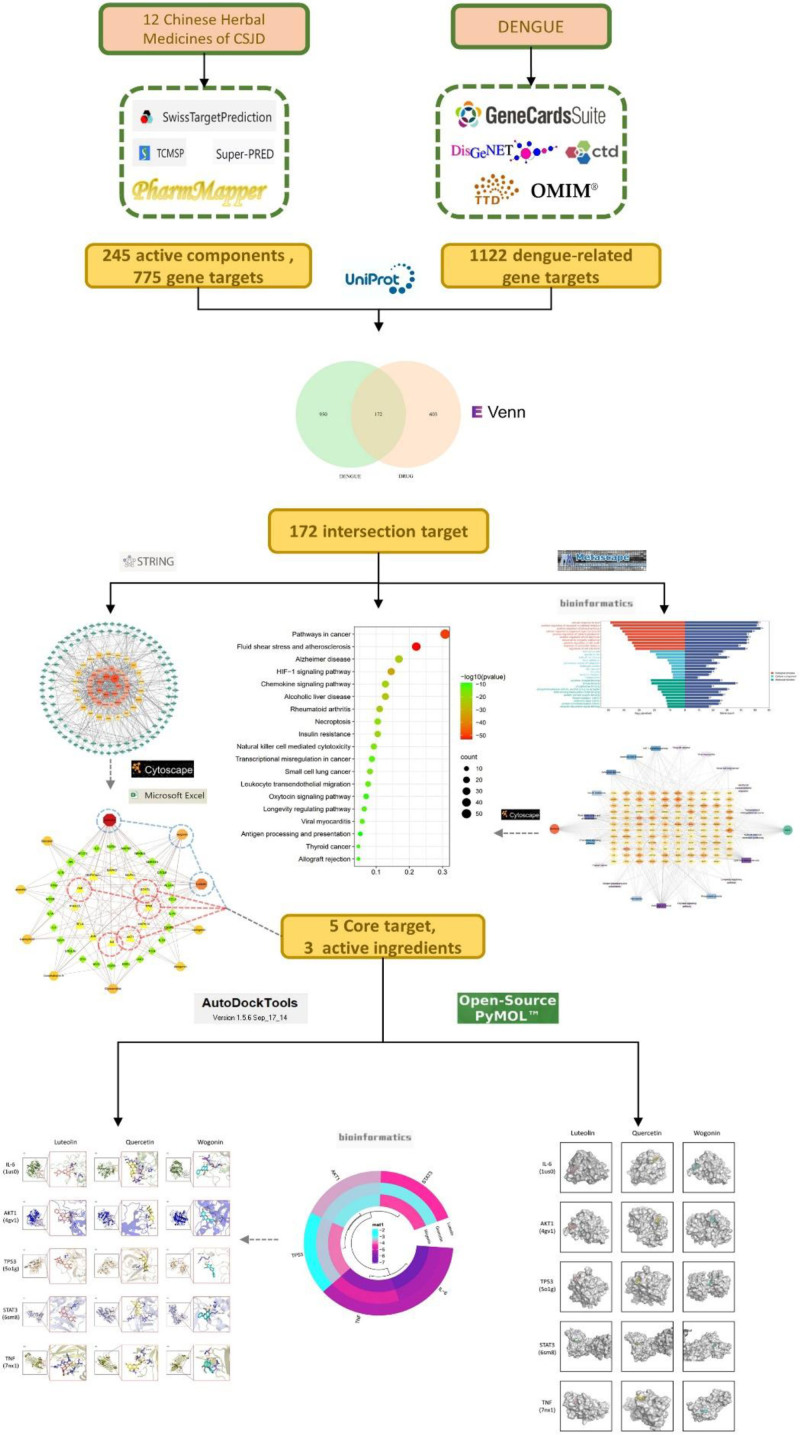
Flowchart of this study. CSJD = Chaishi Jiedu Granules.

At present, there is still a lack of in-depth research on its targets and pathways. Therefore, this study seeks possible targets and potential mechanisms of action of CSJD in the treatment of dengue fever with the help of network pharmacology, so as to provide ideas for the research and development of new dengue drugs in the future and lay a foundation for its clinical application. The flowchart outlining the methodology of this study is displayed in Figure 1.

## 2. Methods

### 2.1. Screening of active constituents and target of CSJD

Traditional Chinese Medicine Systems Pharmacology Database and Analysis Platform (TCMSP)^[11]^ is one of the most commonly used databases for the screening of effective ingredients of traditional Chinese medicine and the acquisition of corresponding targets. The advantage of the TCMSP database is that it provides oral bioavailability (OB) and drug similarity (DL) parameters, which play a critical role in the evaluation of drug efficacy. Only when these two values exceed a certain value (OB ≥ 25% and DL > 0 or OB > 0% and DL ≥ 0.18) can the general properties of the component be effectively reflected.

Through the TCMSP (https://old.tcmsp-e.com/tcmsp.php) database,^[11]^ 12 drugs of CSJD were searched, and the active ingredients and their corresponding targets were collected when OB ≥ 30% and DL ≥ 0.18 were set. The main active ingredients of traditional Chinese medicines (gypsum, pinellia fascia, buffalo horn, talc) not included in the TCMSP database were obtained through literature review.^[12,13]^ The molecular formula of the ingredients retrieved above is obtained through PubChem platform (https://pubchem.ncbi.nlm.nih.gov/).^[14]^ Using TCMSP database, SwissTargetPrediction (http://swisstargetprediction.ch/),^[15]^ the SuperPred3 (https://prediction.charite.de/subpages/target_prediction.php),^[16]^ pharmMapper (http://www.lilab-ecust.cn/pharmmapper/)^[17,18]^ platform for retrieval and prediction to obtain the corresponding targets of active ingredients. All targets of CSJD were imported into UniProt database (https://www.uniprot.org/)^[19]^ for standardized conversion, and corresponding Gene Symbol was obtained. The main target of CSJD was obtained after repeated values were screened out.

### 2.2. Acquisition of disease targets

The gene targets of dengue were collected from 5 databases by querying with the keywords “dengue”: DisGeNET database (DisGeNET, https://www.disgenet.org/)^[20]^; Online Mendelian Inheritance in Man database (OMIM, https://omim.org/); GeneCards Human Gene database (GeneCards, https://www.genecards.org/)^[21]^; Comparative Toxicogenomics Database (CTD, http://ctdbase.org/)^[22]^; Therapeutic target database (TTD, https://db.idrblab.net/ttd/).^[23]^ Then, we removed duplicate targets and merged all collected targets from the 5 databases.

### 2.3. Acquisition of common targets by CSJD and dengue

Dengue targets and CSJD targets import E-Venn platform (EVeen, http://www.ehbio.com/test/venn/#/),^[24]^ CSJD and dengue intersection of targets, and map Wayne.

### 2.4. Protein-protein network construction of intersection targets and core target selection

Import the intersection target into the STRING platform (STRING, https://cn.string-db.org/cgi/input?sessionId=bn13JMrNbvYq&input_page_show_search=on),^[25]^ Set the Organisms to Homo sapiens, set the maximum confidence to 0.9, and then hide the protein in the network that is not associated with other proteins. Protein-protein interaction network (PPI) was obtained and saved as TSV file. Import TSV files into Cytoscope 3.9.1 software, perform Network Analyzer topology analysis, and export the results to Excel 16.0. The 152 targets are arranged in descending order of degree. The median of the degree value of all nodes is 13. Therefore, 47 targets with Degree ≥ 13 were selected, and those with degree values ranging from 11th to 47 were sub-core targets. Take the top 10 targets in degree ranking as the core targets. The core targets were introduced into Cytoscape 3.9.1 to construct the core target interaction network.

### 2.5. Gene ontology (GO) functional analysis and Kyoto encyclopedia of genes and genomes (KEGG) pathway enrichment analysis

The intersection targets were imported into Metascape platform (Metascape, https://www.bioinformatics.com.cn)^[26]^ for KEGG, GO analysis. Species was set as H sapiens, Min Overlap = 3, *P* value < .01, Min Enrichment > 1.5. GO enrichment analysis included biological process (BP), molecular function (MF) and cellular component (CC). The results through the bioinformatics platform (https://www.bioinformatics.com.cn) of visual display.

### 2.6. Molecular docking verification

The degree of PPI network of active ingredient-target was calculated and ranked according to the degree value. Irrelevant ingredients and targets were removed based on literature review, and the top 5 targets were selected for molecular docking with the top 3 active ingredients. Downloaded from TCMSP ingredients of structure, the structure of the key targets from RCSB Protein Data Bank (PDB, https://www.rcsb.org/)^[27]^ to download. The key target was introduced into PyMOL 2.5 for water removal and ligand treatment. The PDB structure of the core target was introduced into the AutoDock 4.2.6, hydrogen was added, and the charge was calculated to determine the rigidity of the atom, and then molecular docking was carried out. The higher the absolute value of the docking fraction, the stronger the binding ability of the small molecule to the protease target. The docking site with the lowest energy was selected and imported into PyMOL 2.5 for visual display.

## 3. Results

### 3.1. Main active constituents and corresponding targets of CSJD

The 12 Chinese herbs of CSJD were retrieved in TCMSP database, and OB ≥ 30%, DL ≥ 0.18 was set, and a total of 267 active ingredients were obtained through literature supplement, and 245 active ingredients were obtained after weight removal. The 245 active ingredients were retrieved and predicted by TCSMSP, Swiss and other platforms. A total of 3889 targets were obtained, and 775 targets were obtained after weight removal.

### 3.2. The main target of dengue fever

Using dengue as the search term, 2 targets were obtained in OMIM, 5 targets in TTD, 923 targets in GeneCards, 110 targets in DisGeNET, and 2684 targets in CTD. A total of 1122 targets were obtained after merging and removing duplicates.

### 3.3. Key targets and construction of PPI network

**Figure 2.**
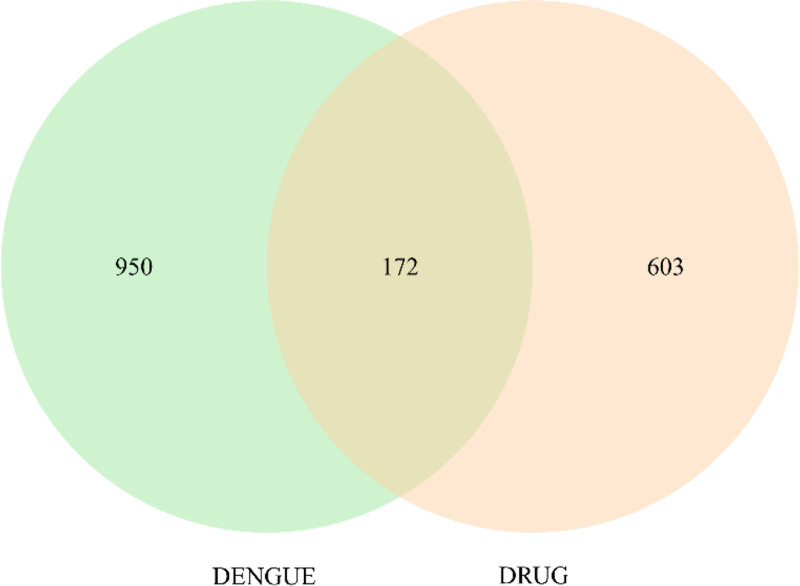
The Venn diagram of CSJD and dengue intersection targets. CSJD = Chaishi Jiedu Granules, DURG = CSJD, DENGUE = dengue.

**Figure 3.**
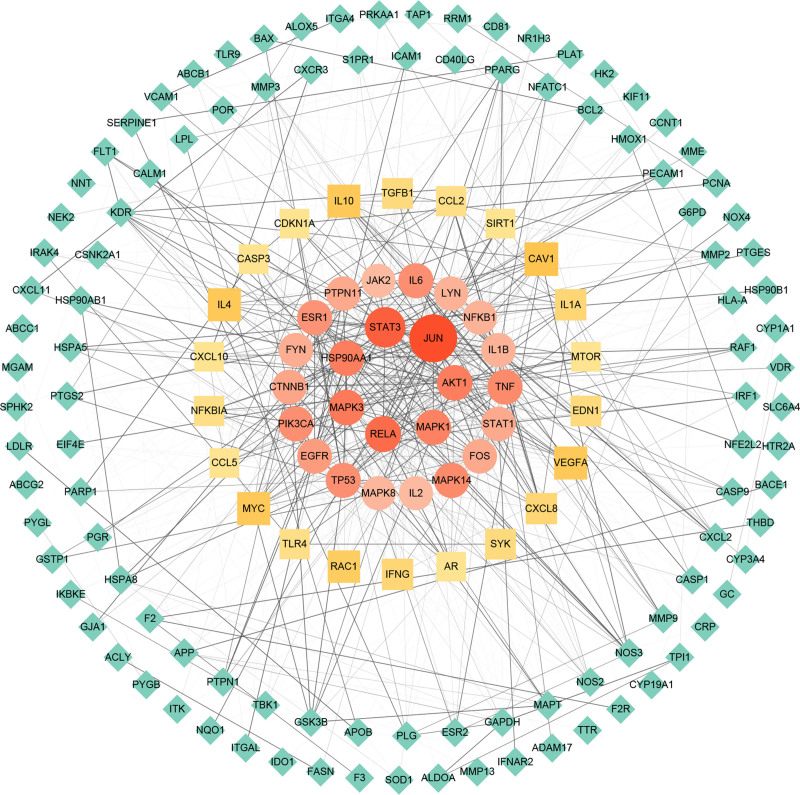
PPI network of the 172 intersection targets of CSJD and DENGUE. Red represents the core targets, yellow represents the sub-core targets, and green represents the intersection targets other than the core and sub-core targets. The darker the color, the higher the degree value. CSJD = Chaishi Jiedu Granules, PPI = protein-protein interaction.

Seven hundred seventy-five drug targets and 1122 disease targets after weight removal were introduced into the Evenn platform, and 172 intersection targets of the 2 were obtained, and the Venn diagram was made. (Fig. 2) 172 intersection targets were imported into STRING database to obtain PPI action network. Furthermore, 47 key targets, including 35 sub-core targets and 12 core targets, were selected by Cytoscape 3.9.1. The intersection target interaction network was mapped by Cytoscape 3.9.1. (Fig. 3)

### 3.4. Key component screening and key component - key target network construction

**Figure 4.**
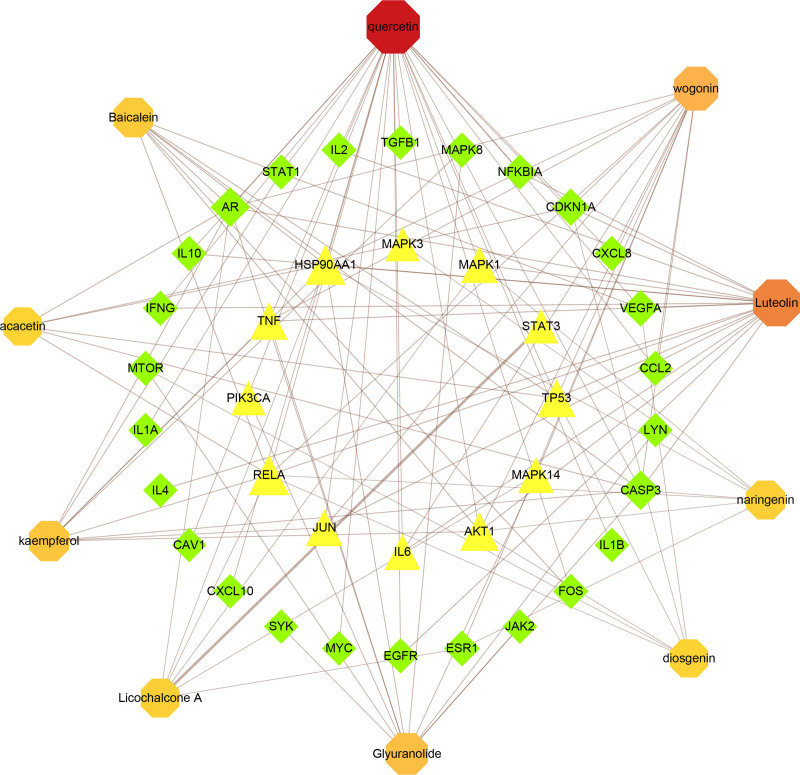
Key component - core target network. The red in the outermost circle represents the 10 core components of CSJD, the yellow in the innermost circle represents the core targets, and the green in the middle represents the sub-core targets. CSJD = Chaishi Jiedu Granules.

Topological analysis was carried out on 215 ingredients of CSJD (excluding 30 ingredients that have no intersection with the intersection targets) and 47 key targets on Cytoscape 3.9.1, and the results were output into Excel table. After ranking by degree, the top 10 ingredients were selected as key ingredients for presentation. In addition, key component - core target network construction is carried out through Cytoscape 3.9.1. (Fig. 4)

### 3.5. GO and KEGG enrichment analysis

**Figure 5.**
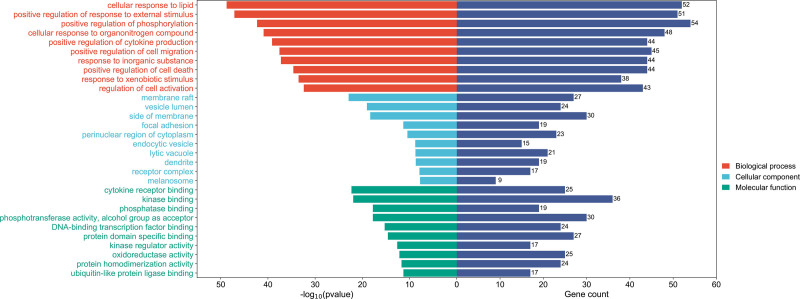
The top 10 ranked processes for BP, CC, and MF. BP = biological process, CC = cellular component, MF = molecular function.

After importing 172 drug-disease intersection targets into Metascape platform for GO enrichment analysis, a total of 2572 targets were obtained. Among them, there are 2235 BP entries, It mainly involves cellular response to lipid, positive regulation of response to external stimulus, positive regulation of phosphorylation, cellular response to organonitrogen compound, positive regulation of cytokine production, positive regulation of cell migration, response to inorganic substance, positive regulation of cell death, response to xenobiotic stimulus and regulation of cell activation. There were 134 CC items, which mainly involved membrane raft, vesicle lumen, side of membrane, focal adhesion, perinuclear region of cytoplasm, endocytic vesicle, lytic vacuole, dendrite, receptor complex and melanosome. There were 203 MF entries, which mainly involved molecular functions such as cytokine receptor binding, kinase binding, phosphatase binding, phosphotransferase, with alcohol group as acceptor, DNA-binding transcription factor binding, protein domain specific binding, kinase regulator activity, oxidoreductase activity, protein homodimerization activity, ubiquitin-like protein ligase binding, etc. The top 10 ranked processes for CC, BP, and MF are shown in Figure 5.

**Figure 6.**
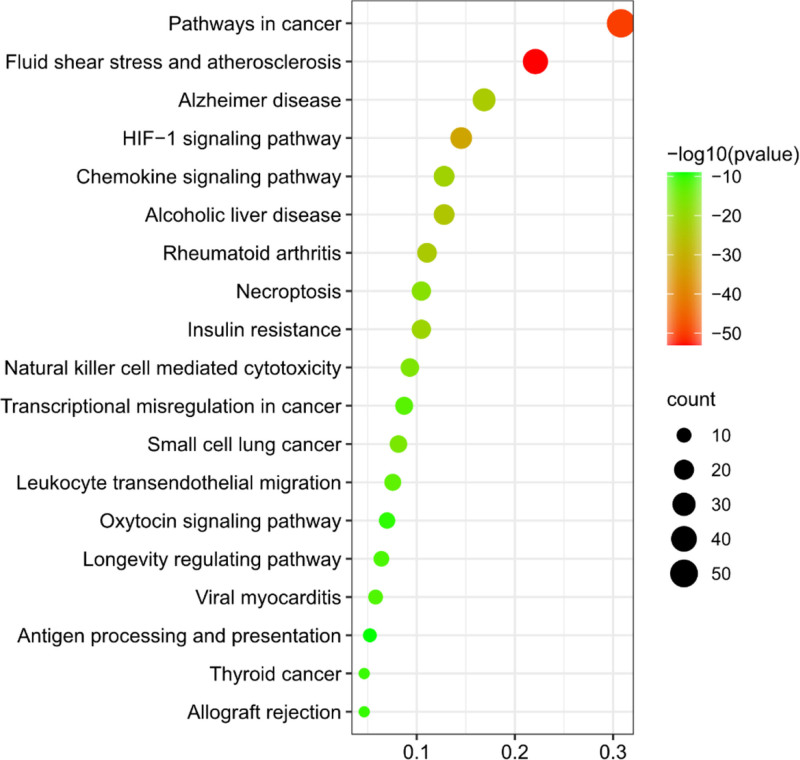
Results of KEGG enrichment analysis. The X-axis represents the significant increase in counts or GeneRatio for these terms. Y-axis represents the top 20 pathways. KEGG = Kyoto encyclopedia of genes and genomes.

**Figure 7.**
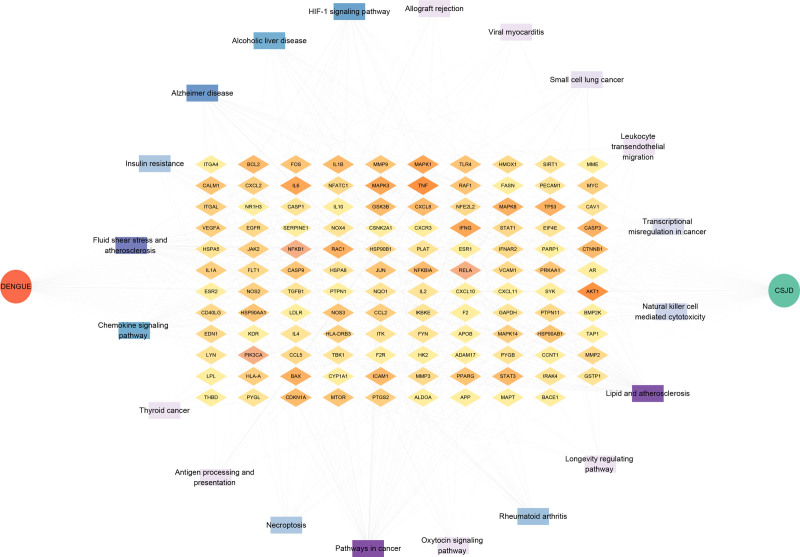
Network diagram of core pathways and intersecting targets. The outer purple circle represents the 20 core pathways, and the inner yellow matrix represents their corresponding core targets. The darker the color, the higher the degree value.

A total of 172 intersection targets were imported into the Metascape platform for KEGG enrichment analysis. With *P* < .01 as the screening condition, 203 pathways were obtained. According to Log10 (P) value, the top 20 critical pathways were obtained by removing irrelevant pathways, which were Lipid and atherosclerosis; Fluid shear stress and atherosclerosis; Pathways in cancer; Hypoxia-inducible factor 1 (HIF-1) signaling pathway; Alcoholic liver disease; Rheumatoid arthritis; Alzheimer disease; Chemokine signaling pathway; Insulin resistance; Necroptosis; Natural killer cell mediated cytotoxicity; Small cell lung cancer; Leukocyte transendothelial migration; Transcriptional misregulation in cancer; Viral myocarditis; Longevity regulating pathway; Thyroid cancer; Allograft rejection; Oxytocin signaling pathway; Antigen processing and presentation. Import the above 20 channels into SRplot platform for mapping. (Fig. 6) Then the top 20 pathways and 172 core targets were imported into Cytoscape 3.9.1 to construct a network diagram of key pathways and core targets (Fig. 7). Based on literature search, the number of key targets contained in the pathway, and the exclusion of low-correlation pathways, 5 key pathways were finally selected, which were respectively Lipid and atherosclerosis; HIF-1 signaling pathway; Chemokine signaling pathway; Leukocyte transendothelial migration; Antigen processing and presentation.

### 3.6. Active ingredient-target molecular docking

**Figure 8.**
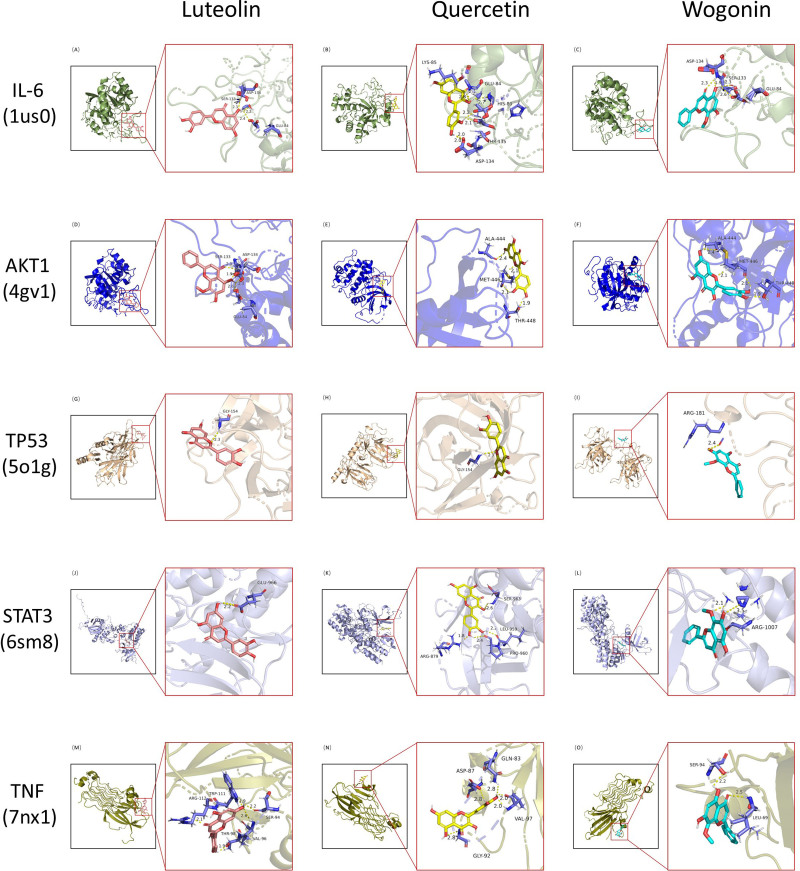
Binding energy heatmap of molecular docking. The darker the color represents, the lower the binding energy.

**Figure 9.**
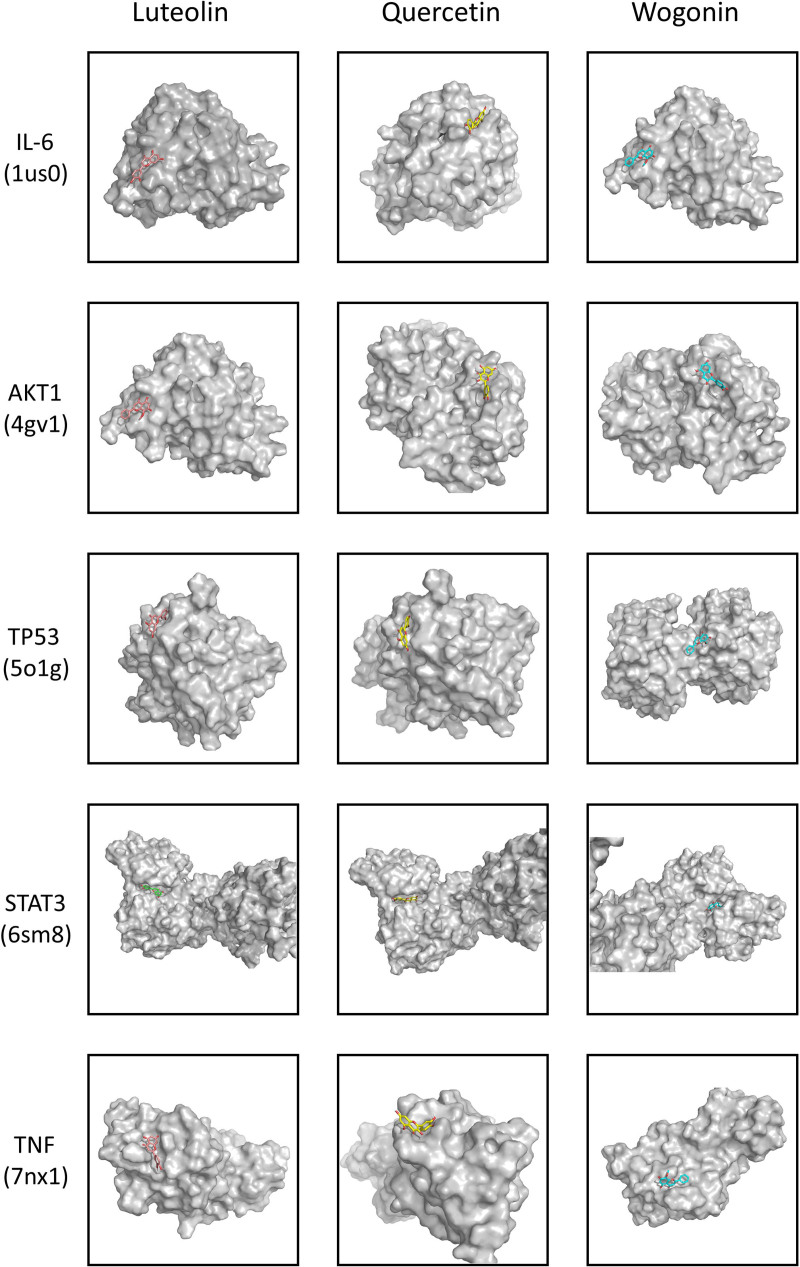
Visualization of 3D molecular docking results.

**Figure 10.**
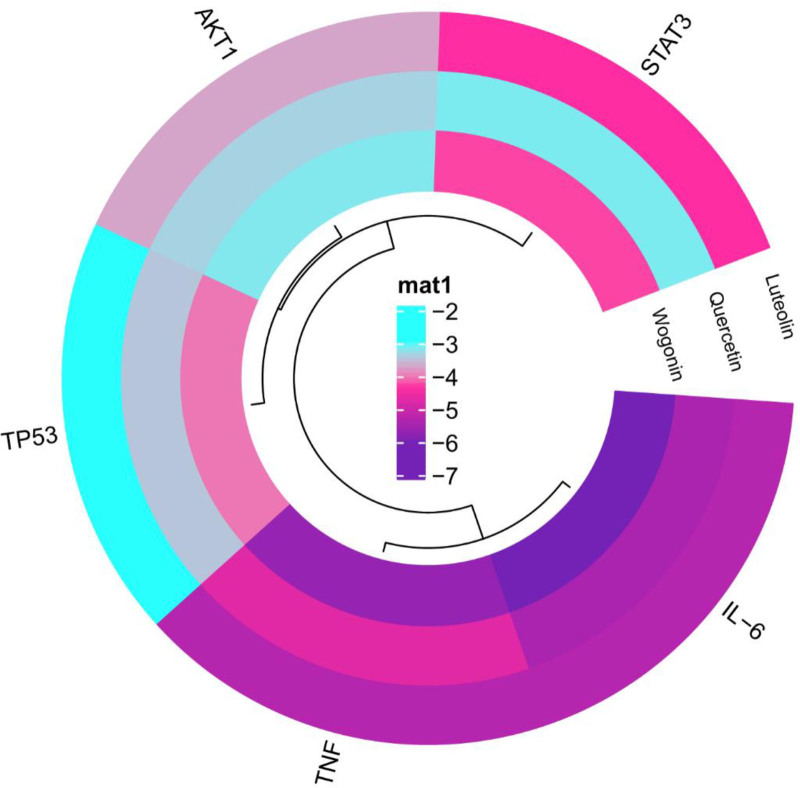
Visualization of 3D molecular docking surface results.

Three active ingredients strongly associated with dengue and 5 targets with a high degree were selected for molecular docking through literature review. The 3 active ingredients were luteolin, quercetin and wogonin. The 5 core targets were IL-6, AKT1, TP53, STAT3, and TNF. The results showed that in the docking results of AutoDockTools 1.5.7 (ADT), all active ingredients could form docking models with core proteins. (Fig. 8) Table 1 presents the binding energies of key components and key targets.(Table 1) The lowest binding energy of IL-6 with luteolin, quercetin and wogonin and the binding energy of TNF with luteolin and wogonin were lower than -5. The lowest binding energy of AKT1 with luteolin, quercetin, wogonin and STAT3 with luteolin, quercetin, wogonin is less than -3. In addition, TP53 can form a stable docking model with luteolin, quercetin and wogonin. (Figs. 9 and 10)

**Table 1:** Statistical results of molecular docking binding energy (binding energy/kcal mol^−1^).

	Luteolin	Quercetin	Wogonin
IL-6	−5.33	−5.48	−6.08
AKT1	−3.73	−3.36	−3.18
TP53	−2.97	−3.47	−4.04
STAT3	−4.35	−3.15	−4.28
TNF	−5.32	−4.76	−5.72

## 4. Discussion

Clinical symptoms of Dengue fever range from mild fever to severe Dengue hemorrhagic fever or Dengue shock syndrome, accompanied by thrombocytopenia, leukopenia, and increased vascular permeability. While primary infection can activate the immune response against the Dengue virus serotype, the severity of the disease is increased through heterotypic infections of various serotypes and antibody-dependent enhancement (ADE).^[28]^ Dengue fever has caused a significant burden on the global economy and healthcare system, with researchers investing considerable effort and resources into the development of Dengue fever drugs. However, no specific drugs have been found to date, and clinical support for symptomatic treatment remains the primary approach.

Dengue fever presents symptoms characteristic of acute infectious diseases, including biphasic fever, myalgia, headache, joint pain, retro-orbital pain, rash, thrombocytopenia, lymphadenopathy, and leukopenia.^[29]^ Dengue fever has 3 distinct stages: the febrile phase, the critical phase, and the recovery phase. The febrile phase is characterized by sudden high fever and dehydration that can last for 2 to 7 days. The recovery phase is characterized by the resolution of symptoms, observed with rash, itchiness, and increased appetite. Dengue fever falls under the “plague” category in TCM, and can be studied through differential diagnosis and treatment of “Yizhen” and “Shiwen” in Warm Disease theory. Experiments by Tang and others have shown that TCM differential diagnosis and treatment of Dengue fever patients can improve their symptoms and shorten fever time.^[30]^ Experiments by Zhou Wen and others indicated that traditional Chinese medicine can effectively improve the clinical efficacy of Dengue fever patients, increase white blood cell and lymphocyte counts in patients, and improve their clinical symptoms and signs.^[31]^ CSJD has been clinically applied in China and Southeast Asian countries (such as Cambodia and Pakistan) since 2014. It has been observed to significantly alleviate fever, relieve pain and joint pain, shorten the fever course of Dengue fever patients, improve their clinical symptoms and signs, increase the efficacy when combined with conventional treatment, and shorten the hospitalization time of patients.^[5]^ Animal experiments have also confirmed its efficacy in relieving symptoms and promoting the recovery of Dengue fever patients.^[6]^ CSJD is a unique combination of White Tiger Decoction and Liu Yi San with specific modifications. Its treatment principles revolve around clearing heat, resolving dampness, replenishing qi, and nourishing Yin. Radix Bupleuri and Gypsum are the chief herbs with Radix Bupleuri providing heat-relieving effects and promoting the smooth flow of Qi while preventing thermal stasis. Meanwhile, Gypsum provides cooling effects and clears heat and dampness. Talc and Coix seed promote diuresis and relieve dampness, Scutellaria clears heat, and dries dampness, and Pueraria relieves the muscles, reduces fever, and promotes the eruption. Adjuvant herbs like Buffalo Horn, Codonopsis, Siphonostegiae Herba, and Arum Ternatum Thunb all play a role in clearing heat and cooling blood, promoting Qi and nourishing Yin, stopping pain, promoting diuresis and relieving dampness, and coordinating the formula respectively. Finally, Glycyrrhizae Radix Et Rhizoma is used to harmonize the formula. The general application of this formula, in combination with the 4 methods of clearing, scattering, harmonizing, and replenishing, aims to clear heat, resolve dampness, cool blood, detoxify, and nourish Yin. Ultimately, it restores smooth circulation of Qi and blood, balances Yin and Yang, and achieves the effects of reducing fever, resolving dampness, external scouring, and internal transformation.

This study utilized network pharmacology and literature retrieval methods to identify 10 key ingredients of CSJD that are effective against Dengue fever, namely luteolin, kaempferol, quercetin, licochalcone A, baicalein, glyuranolide, naringenin, acacetin, wogoni, and diosgenin. Luteolin, primarily found in Arum Ternatum Thunb and Licorice, exerts its anti-Dengue virus effect by interfering with the host Furin protein and its interaction with the viral prM and NS2B proteins.^[32]^ In addition, luteolin also reduces the level of IL-6 released by Peripheral Blood Mononuclear Cells infected with Antibody-Dependent Enhancement of Dengue Virus (ADE-DENV).^[33]^ Luteolin can reduce both the viral viremia levels and the release of inflammatory cytokines associated with Dengue virus infection. Quercetin, found in Radix Bupleuri and Licorice, has also shown promise in anti-Dengue virus research. Scutellariae Radix, a component of the CSJD formula containing baicalin, is also reported to exhibit effective in vitro anti-Dengue activity. Scutellariae Radix metabolite baicalein has been shown to inhibit Dengue virus replication.^[34–36]^ Naringin, found in Licorice, has inhibitory effects on Dengue virus infection and replication in in vitro experiments, as does sophoricoside, found in Scutellariae Radix. Overall, these findings suggest that these key ingredients of CSJD exert their anti-Dengue virus effects through a diverse range of mechanisms.^[37]^

The 12 key targets selected in this study were JUN, STAT3, relA, MAPK3, AKT1, HSP90AA1, MAPK1, MAPK14, TNF, TP53, PIK3CA, and IL-6. They are involved in cell metabolism, proliferation, transcription, inflammatory response, cell survival, growth and angiogenesis, inhibition of viral replication and other processes. Studies have suggested that serum IL-6 levels detected in dengue patients are significantly increased.^[38]^ IL-6 is an inflammatory cytokine, and its release is related to the activation of inflammatory response and immune damage. CSJD may interfere with inflammation by releasing IL-6 factor, thus achieving the purpose of treating dengue fever. AKT1 is one of the genes related to autophagy expression. CSJD may inhibit the occurrence of autophagy by down-regulating the expression of AKT1 by targeting, thus hindering the replication of dengue virus.^[39]^ STAT3 is a key transcription factor mediating the early response of dengue shock syndrome. Relevant studies have suggested that the decrease of STAT3 level can lead to a significant decrease in the expression and replication of dengue virus protein, and CSJD may inhibit the expression and replication of Dengue virus by reducing the level of STAT3 in patients.^[40]^ Related studies have shown that the transcription factor p53 mediated by the TP53 gene inhibits Dengue virus replication by activating the type I interferon pathway. Dengue virus can inhibit the production of white blood cells and platelets in the bone marrow of patients, resulting in leukopenia and thrombocytopenia.^[41]^ TNF, also known as tumor necrosis factor, has been linked to bleeding in patients during dengue infection.^[42]^ CSJD may regulate the level of TNF in vivo to control bleeding and thus improve patients symptoms.

Twelve key pathways were identified by Metascape platform and literature review, among which the pathways most closely associated with dengue were Lipid and atherosclerosis; HIF-1 signaling pathway; Chemokine signaling pathway; Leukocyte transendothelial migration; Antigen processing and presentation. Lipids are the structural and functional basis of biofilms (subcellular) and are involved in a variety of physiological and disease processes. Lipid metabolism has been proved to play an important role in the entry, replication, assembly and secretion of various viruses. Blood tests in patients with severe dengue often show low levels of high-density lipoprotein, and lipid raft depletion mediated by ApoA1, a major structural protein of high-density lipoprotein, inhibits DENV from attaching to cell surfaces.^[43]^ The study of Xiao et al^[44]^ indicated that lipid metabolism disorder is a typical clinical feature of dengue virus infection, and the interaction between DENV and lipid metabolism is involved in viral replication and pathogenesis. In addition, studies have reported that statins and lipid-regulating drugs can play an anti-dengue virus role, indicating that lipid metabolism plays a certain role in viral infection.^[45]^ By querying the pathway on the Kyoto Encyclopedia of Genes and Genomes platform (KEGG, https://www.genome.jp/kegg/), it is known that key targets such as PIK3CA, AKT1, TNF, IL-6, and other targets participate in the pathway of lipid and atherosclerosis. Therefore, CSJD may regulate targets such as PIK3CA, AKT1, TNF, IL-6, and others through the lipid and atherosclerosis pathway, block the key pathways and molecules of lipid metabolism, and thus achieve the effect of inhibiting dengue virus replication and improving clinical symptoms of patients.

HIF-1 is a major regulatory factor in cellular response to hypoxia and a key metabolic regulatory factor in response to hypoxia stress at the molecular level.^[46]^ The protein expression induced by HIF mainly helps the metabolism and survival of hypoxic cells, and plays a role in assisting angiogenesis, erythropoiesis, apoptosis, cell differentiation/survival, glucose metabolism, pH regulation and iron metabolism. HIF-1 is composed of α-subunit with catalytic activity and structurally expressed β-subunit, and its HIF-1α is the main regulatory factor of cell response to hypoxia.^[47]^ Under normal oxygen concentration conditions, HIF-1α was rapidly degraded. In anoxia, the expression can be stabilized to enhance the adaptability of cells to hypoxia. Some studies have shown that Dengue virus-induced hypoxia and metabolic changes can enhance viral RNA replication, and the mechanism is related to the increased expression of HIF-1α/2α and AKT, and the increase of anaerobic glycolysis.^[48]^

Transendothelial migration is the migration of white blood cells from blood to tissue across the vascular endothelium and is critical for immune surveillance and inflammation. This process helps to control early infection events.^[49]^ The inflammatory immune response requires recruitment of white blood cells to the site of inflammation in response to foreign injury. Chemokines are small chemoattractant peptides that provide directional cues for cell transport and are therefore critical for protective host responses. In addition, chemokines regulate a large number of biological processes in hematopoietic cells to guide cell activation, differentiation, and survival. CSJD may regulate inflammation and immune response through chemokine signaling pathway, thus achieving its therapeutic effect on dengue fever. DENV evades the host adaptive immune response by antigenic variation, ADE, partial maturation of prM proteins, and inhibition of antigen presentation.^[50]^ CSJD may inhibit the virus by enhancing the body’s immune response through antigen processing and presentation pathways.

Finally, in order to further verify the binding ability of CSJD core active ingredients with molecular targets, we verified the docking verification of the top 3 active ingredients with the top 5 target proteins through molecular docking technology. The results showed that luteolin, quercetin and wogonin could form stable docking models with IL-6, AKT1, TP53, STAT3, and TNF, respectively. From the visualization of molecular docking surface, it can be observed that all 3 key ingredients are in contact with the active pockets of the 5 key targets. (Fig. 9) These results suggest that the combination of these active ingredients with core targets plays an important role in the treatment of dengue by CSJD.

## 5. Conclusion

In summary, our findings suggest that CSJD comprises key ingredients such as luteolin, quercetin, baicalin, and naringenin, which act on critical targets such as JUN, STAT3, relA, MAPK3, AKT1, MAPK1, MAPK14, TNF, TP53, PIK3CA, and IL-6. By regulating signaling pathways such as those related to lipid and atherosclerosis pathway, HIF-1 signaling pathway, chemokine signaling pathway, leukocyte migration through endothelia, antigen processing and presentation, CSJD may play an important role in inhibiting dengue virus replication, enhancing anti-inflammatory responses, improving immunity, regulating coagulation and fibrinolysis, and modulating energy and lipid metabolism. Nonetheless, the limitations of network pharmacology necessitate further experimental validation of these preliminary results.

## Author contributions

**Investigation:** Cong Li.

**Methodology:** Cong Li.

**Project administration:** Cong Li.

**Software:** Sanqi Huang.

**Supervision:** Luping Lin, Yexiao Tang.

**Writing – review & editing:** Luping Lin.
